# Debt portfolios of street vendors: Survey data from Colombia

**DOI:** 10.1016/j.dib.2019.103714

**Published:** 2019-03-15

**Authors:** Lina M. Martinez, Juan David Rivera-Acevedo

**Affiliations:** a Universidad Icesi, Cali, Colombia; b Erfurt University, Erfurt, Germany

## Abstract

This paper presents data about the indebtedness of the poor, in particular, street vendors from Cali, Colombia. In Latin America, as in many other developing regions, the poor have no access to credit from regulated institutions. Bank fees, transaction costs and lack of durable assets to back up indebtedness, exclude the poor form the banking system and their only resort for credit are moneylenders who charge predatory interest rates. In order to assess the economic implications of payday loans, information about indebtedness was collected amongst street vendors in Cali, Colombia. A random sample of 300 street vendors was surveyed at two large street vending sites in the city in 2016. Respondents were inquired about income, expenses, household composition, and access to banking services, credit, and indebtedness. This data in brief presents the value of the gathered information, the general characteristics of this research and the methodology used. Data of this manuscript is associated with the publication Martinez, L., & Rivera-Acevedo, J. D. (2018).

**Table:** Specifications table

Subject area	Public Policy
More specific subject area	*Microfinance*
Type of data	Text, dummy and metric variables
How data was acquired	Survey data
Data format	Raw
Experimental factors	There was not random assignment component in data in this manuscript
Experimental features	There was not an experimental component in the data set
Data source location	Cali – Colombia
Data accessibility	Observatorio de Políticas Públicas – POLIS www.icesi.edu.co/polis/
Related research articles	Martinez, L., & Rivera-Acevedo, J. D. (2018). Debt portfolios of the poor: The case of street vendors in Cali, Colombia. *Sustainable Cities and Society*, *41*, 120–125.

**Table:** 

**Value of the data** • There is a lack of information about the link between poverty and access to regulated credit. The information gathered from this study allows an assessment of the economic consequences of credit in form of payday loans amongst the poor and contributes to a better understanding of this phenomenon. • Data was collected amongst street vendors, a population that is poor and vulnerable, but has a larger income than the average citizen in Cali (Martínez, et al, 2017). Linking the information between poverty, income and lack of access to credit, allows a better understanding of different mechanisms that perpetuate poverty. • This data is highly relevant for policy making purposes. Most of the government interventions aimed at providing regulated credit to the poor have focused on microfinance loans. However, microfinance credits do not take into account the dynamics and behaviors of the poor in terms of savings and repayment capabilities. Most of the credit programs promoted by the government are not tailored for the needs and possibilities of the poor. • Data from this study can be linked with several observable characteristics of informal workers like access to welfare programs and demographic information. By linking this data with broader studies it is possible to draft conclusions for larger populations that earn their income through the informal economy and are at the bottom of the pyramid.

## 1 Data

Information about the debt portfolios of the poor was collected in Cali, Colombia. Data were collected through a structured survey. Informants were 300 street vendors randomly selected at two street vending sites in the city. Field work was conducted between January and February 2016. Table 1 presents general characteristics of the surveyed population.

**Table 1:** Indebtedness information of street vendors in Cali, Colombia.

Male	54,6
Average age (years)	42
***Education***	
No education	5,3
Elementary school (%)	32
Incomplete high school (%)	29,3
High school diploma (%)	26,3
***Indebtedness***	
Has someone to borrow money from (%)	67,3
Has had any type of loan in the past (%)	74
Has had more than one loan at the same time (%)	27
Currently paying a loan (%)	43
Average time for loan repayment (months)	13, 1
**In debt by lender**	
Family (%)	4,4
Friend (%)	3,5
Bank (%)	16,8
Microfinance institution (%)	23,9
Payday-loan (%)	51.3
**Average interest rates per month**	
Family (%)	0
Friend (%)	10
Bank (%)	3
Microfinance institution (%)	2,5
Payday-loan (%)	20,4
N. Observations	300

### 1.1 Experimental design, materials and methods

**Fig. 1:**
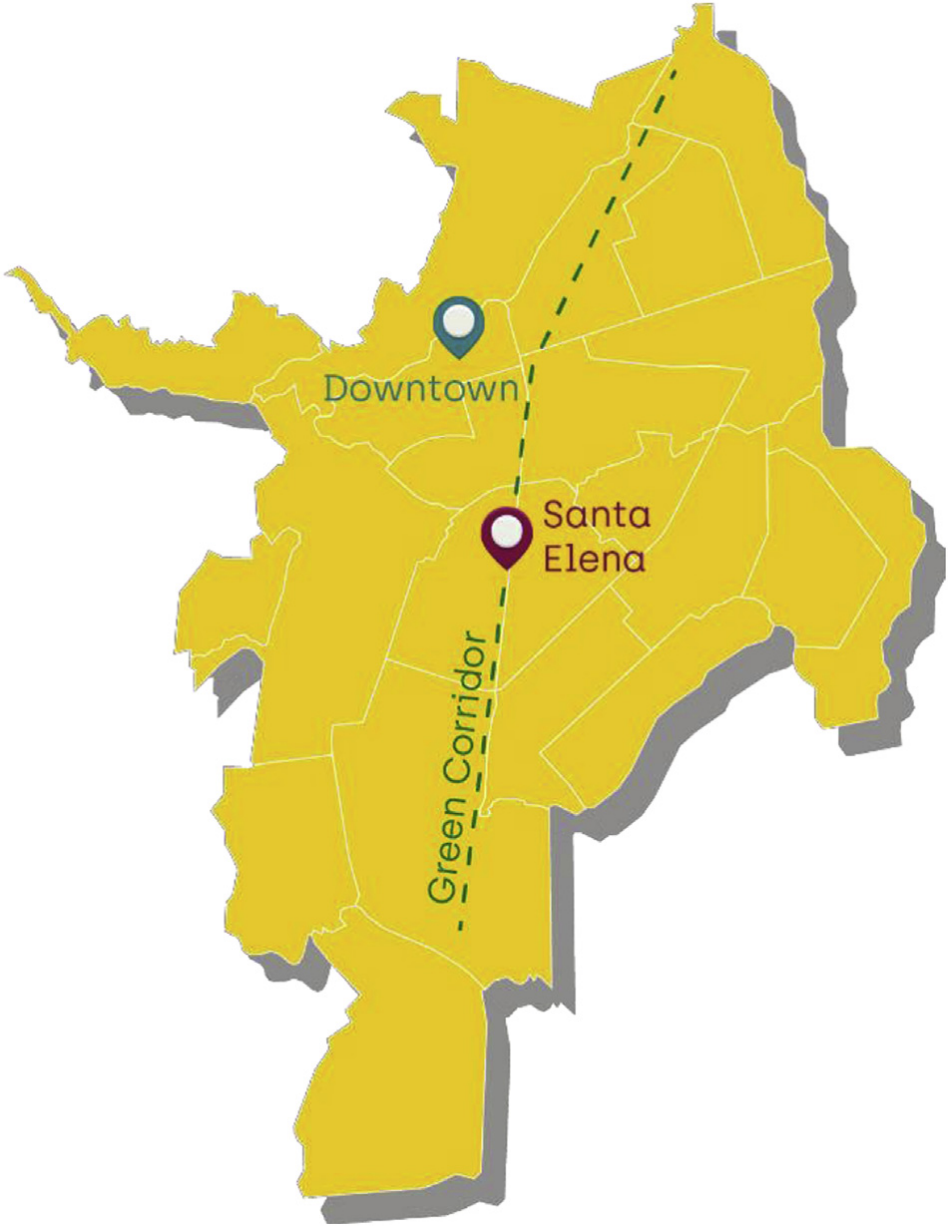
Field work locations: Street vending sites in Cali, Colombia.

Based on the information of the planning department of Cali, there are nine street vending sites in the city. Information presented in this analysis was collected at the two largest sites: Downtown and Santa Helena. The downtown site is located in the heart of the city where most of the government offices are situated. Street vendors occupy an area of about 13 blocks that provides a vast range of informal and formal activities. Santa Elena is a food market that covers about 12 blocks. It is located next to an area with high criminal activity and lies in the middle of an urban renovation plan by the government called the “green corridor”. Fig. 1 presents the locations where the field work was conducted.

For data collection, the authors designed a structured survey (Supplemental material). The questionnaire was piloted with 15 street vendors at other sites -different from Downtown and Santa Helena-to verify the clarity of the questions and the general structure of the questionnaire. Minor changes were introduced in the final questionnaire after the pilot. Trained pollsters collected the information alongside two field supervisors. Respondents were selected randomly and pollsters provided an explanation of the purpose of the study. Anonymity was guaranteed and it was made clear that the information would be used for academic purposes only. Participation was voluntary and respondents could stop the survey at any time. We collected 300 complete surveys and based on this sample, is not possible to generalized on the indebtedness of all street vendors in the city. Few respondents quitted after the survey started.

Respondents were asked 66 questions about demographic information (age, gender, and education), indebtedness, income, expenses, type of sales, and expectations of their economic future. The uninterrupted survey took about 20 minutes, but since the participants usually continued their work it took about 40 minutes in most cases. The information collected can be used for descriptive analysis aimed at informing policymakers and academics about the lack of financial inclusion and indebtedness of the of street vendors.

This study follows local and international rules for empirical research. Likewise, respondents provided verbal consent before survey commencement. The survey did not inquire about personal information that allows the identification of any informant. Information about this study is available at: www.icesi.edu.co/polis/.
