# Supplementary material for: Debt portfolios of street vendors: Survey data from Colombia
Source: Data Brief. 2019 Mar 15;24:103714. doi: 10.1016/j.dib.2019.103714 (PMC6484363; doi:10.1016/j.dib.2019.103714)
Supplement: Multimedia component 1 [file mmc1.pdf]

Conflict of Interest Statement

Manuscript title: Debt portfolios of the poor: Survey Data from Street Vendors in Cali, Colombia

The authors whose names are listed immediately below certify that they have NO affiliations with or involvement in any organization or entity with any financial interest (such as honoraria; educational grants; participation in speakers' bureaus; membership, employment, consultancies, stock ownership, or other equity interest; and expert testimony or patent-licensing arrangements), or non-financial interest (such as personal or professional relationships, affiliations, knowledge or beliefs) in the subject matter or materials discussed in this manuscript.

Author names:

Lina Martínez

Juan David Rivera-Acevedo

This statement is signed by all the authors to indicate agreement that the above information is true and correct

Name	Signature	Date
Lina Martinez	[illegible]	Nov 27-2017
Juan David Rivera A.	Juan David Rivera A.	Nov. 27. 2017.
