# Supplementary material for: Debt portfolios of street vendors: Survey data from Colombia
Source: Data Brief. 2019 Mar 15;24:103714. doi: 10.1016/j.dib.2019.103714 (PMC6484363; doi:10.1016/j.dib.2019.103714)
Supplement: Multimedia component 3 [file mmc3.pdf]

UNIVERSIDAD ICESI — POLIS

Sub-sample survey street vendors downtown and Santa Elena
Access to credit and financial services costs - 2016

**Application date**	**Month**		**Day**		**Survey zone**	**SE**		**Downtown**
**Address:**					**Interviewer's name:**			

1. Year of Birth

____

2.Gender

1 [ ] Male
0 [ ] Female

3. What is the highest educational level you have attained (even if you didn't finish) and the last grade approved in this level?

1 [ ] None
2 [ ] Preschool
3 [ ] Incomplete primary ____
4 [ ] Complete primary ____
5 [ ] Incomplete secondary ____
6 [ ] Complete secondary ____
7 [ ] Technical or technology ____
8 [ ] Under-graduate degree ____
9 [ ] Post-graduate degree ____

4. Currently, do you contribute to pension programs?

1 [ ] Yes
0 [ ] No

5. Do you have anyone to lend you money?

1 [ ] Sí
0 [ ] No

6. Have you made any kind of loan?

1 [ ] Yes 6.1 How many? ____
0 [ ] No (Go to p14)

7. With whom did you make the loan and what is the interest rate? (MULTIPLE CHOICE)

	Interest rate		Interest rate
1 [ ] Family	______	4 [ ] Payday loans	______
2 [ ] Friend	______	5 [ ] Microfinance institution	______
3 [ ] Bank	______	6 [ ] Other ______	______

8. Have you had several loans at the same time?

1 [ ] Yes
8,1 How many?______
0 [ ] No

9. Are you currently paying any loan?

1 [ ] Yes
9,1 How many?______
0 [ ] No (Go to p12)

10. With whom did you make the loan and what is the interest rate? (multiple choice)

	Interest rate		Interest rate
1 [ ] Family	______	4 [ ] Payday loans	______
2 [ ] Friend	______	5 [ ] Microfinance institution	______
3 [ ] Bank	______	6 [ ] Other______	______

11. How much money intended for payment of loans?

____

1 [ ] Daily
2 [ ] Weekly
3 [ ] Biweekly
4 [ ] Monthly

12. On average how long does it take to pay a loan?

______
1 [ ] Days
2 [ ] Months
3 [ ] Years

13. What were the goals of the loans made? (Multiple choice)

1 [ ] Business
2 [ ] Debts
3 [ ] Family
4 [ ] Free investment
5 [ ] Other ______

14. Have you ever applied for a loan at a bank or microfinance institution?

1 [ ] Yes (Go to p15)
0 [ ] No (Go to p16)

15. When you applied for the credit, did you get it?

1 [ ] Yes ⟶ 15.1.2 Loan amount ______
15.1.2 If you are still paying, how long does it take? ______
15.1.3 If you already paid it, how long did it take to pay? ______

2 [ ] No ⟶ 15.2.1 ¿Cuál fue la razón? ______

16. Are you reported in credit - data?

1 [ ] Yes
0 [ ] No

17. In how much would you sell your business, Approximately?

Value ______
1 [ ] Would not sell it 0 [ ] DK

18. If you had the opportunity, where would you like to start a business in Cali?

______

19. What is the best interest rate to access a loan that allows you to expand your business?

______

1 [ ] Daily
2 [ ] Weekly
3 [ ] Monthly
4 [ ] Yearly

20. Type of sale

1 [ ] Meat	11 [ ] Meal	21 [ ] Miscellany
2 [ ] Poultry (chicken, egg)	12 [ ] Confectionery and cigarettes	22 [ ] Porcelains
3 [ ] Fish Shop	13 [ ] Drinks and juices	23 [ ] Lottery/Chance
4 [ ] Fruits	14 [ ] Grocery store	24 [ ] Newspapers, magazines, books
5 [ ] Vegetables	15 [ ] Supermarket	25 [ ] Bags / belts / leather goods
6 [ ] Legume (beans, lentils)	16 [ ] Footwear	26 [ ] Mobile phone accessories
7 [ ] Tuber (potato, yucca)	17 [ ] Clothing store	27 [ ] PC accessories
8 [ ] Medicinal herbs	18 [ ] Glasses	28 [ ] Watchmaking and / or watch repair
9 [ ] Dairy products (milk, yogurth, cheese)	19 [ ] Minutes	29 [ ] Tools
10 [ ] Restaurant/Bakery/Cafeteria	20 [ ] CD´S	30 [ ] Other, which one? ______
